# Mendelian randomization study implicates inflammaging biomarkers in retinal vasculature, cardiovascular diseases, and longevity

**DOI:** 10.1126/sciadv.adu1985

**Published:** 2025-10-24

**Authors:** Ana Villaplana-Velasco, Nicolas Perrot, Yu Hang, Michael Chong, Emanuele Trucco, Muthu R. K. Mookiah, Walter Nelson, Jeremy Petch, Hertzel C. Gerstein, Parminder Raina, Salim Yusuf, Miguel O. Bernabeu, Albert Tenesa, Konrad Rawlik, Guillaume Pare, Alexander Doney, Erola Pairo-Castineira, Marie Pigeyre

**Affiliations:** ^1^ Baillie Gifford Pandemic Science Hub, Centre for Inflammation Research, The Queen’s Medical Research Institute, University of Edinburgh, Edinburgh, UK.; ^2^ Roslin Institute, University of Edinburgh, Edinburgh, UK.; ^3^ Population Health Research Institute, David Braley Cardiac, Vascular and Stroke Research Institute, Hamilton Health Sciences and McMaster University, Hamilton, Ontario, Canada.; ^4^ Department of Pathology and Molecular Medicine, McMaster University, Michael G. DeGroote School of Medicine, Hamilton, Ontario, Canada.; ^5^ Thrombosis and Atherosclerosis Research Institute, David Braley Cardiac, Vascular and Stroke Research Institute, Hamilton, Ontario, Canada.; ^6^ VAMPIRE project, Computing School of Science and Engineering, University of Dundee, Dundee, UK.; ^7^ Centre for Data Science and Digital Health, Hamilton Health Sciences, Hamilton, Ontario, Canada.; ^8^ Institute for Health Policy, Manage and Evaluation, University of Toronto, Ontario, Canada.; ^9^ Department of Medicine, McMaster University, Hamilton, Ontario, Canada.; ^10^ Department of Health Research Methods, Evidence, and Impact, McMaster University, Hamilton, Ontario, Canada.; ^11^ McMaster Institute for Research on Aging (MIRA), McMaster University, Hamilton, Ontario, Canada.; ^12^ Usher Institute, University of Edinburgh, Edinburgh, UK.; ^13^ Bayes Centre, College of Science and Engineering, University of Edinburgh, Edinburgh, UK.; ^14^ Population Health and Genomics, University of Dundee, Dundee, UK.

## Abstract

With the increasing proportion of elderly individuals, understanding biological mechanisms of aging is critical. Retinal vascular complexity, measured as fractal dimension (*D*_f_) from fundus photographs, has emerged as a vascular aging indicator. We conducted a genome-wide association study of *D*_f_ on 74,434 participants from the Canadian Longitudinal Study on Aging, Genetics of Diabetes Audit and Research in Tayside Scotland, and UK Biobank cohorts. We identified a novel locus near *DAAM1*. We found negative genetic correlations between *D*_f_ and cardiovascular disease, stroke, and inflammation but a positive correlation with life span. By combining the genetic determinants of 1159 circulating proteins from the Prospective Urban and Rural Epidemiological cohort with those of *D*_f_ using Mendelian randomization, we identified eight causal mediators, including MMP12 and IgG–Fc receptor IIb, which link higher inflammation to lower *D*_f_, increased cardiovascular disease risk, and shorter life span. These results extend our understanding of the biological pathways underlying aging processes and inform targets to prevention and treatment.

## Teaser

MMP12 and IgG–Fc receptor IIb link higher inflammation to lower *D*_f_, increased cardiovascular disease risk, and shorter life span.

## INTRODUCTION

Understanding the biological pathways that influence aging has become a public health priority (*1*). Aging is associated with an incremental loss of complexity in the dynamics of many physiological systems, rendering them less resilient to environmental stresses (*2*). This loss of resilience progressively increases the vulnerability to late-onset diseases, disability, frailty, and ultimately leads to the death (*3*). Thus, a biomarker signature for “systemic complexity” could potentially provide an indicator of global system resilience against aging.

The retinal vasculature offers a conveniently accessed and imaged perspective on the circulatory system of the human body, providing valuable insights into the vascular health not only of the retina itself but also of other organs (*4*). The automated quantification of density and overall complexity of the retinal vasculature from retinal photographs—achieved through the measurement of its vascular fractal dimension (abbreviated as *D*_f_), with a higher value reflecting a more complex branching pattern—offers a powerful tool in this regard (*5*). Early evidence suggested that aging-related changes in the retinal vasculature result in a decrease of the *D*_f_ measures (*6*). More recently, research has provided compelling evidence that reduced retinal vascular *D*_f_ was associated with various age-related diseases, including hypertension, type 2 diabetes (T2D), coronary artery disease, stroke, and cognitive decline (*5*, *7*). Those findings underscore the clinical significance of assessing retinal vasculature complexity through its *D*_f_, which serves as a valuable indicator of aging-related vascular branching changes. Additionally, *D*_f_ may represent a potential druggable phenotype for therapeutic strategies aimed at modulating vascular aging and mitigating age-related cardiometabolic diseases. However, the causal relationships between *D*_f_ and cardiometabolic diseases, as well as the directionality of these relationships, require further investigation. Moreover, the underlying biomolecular mechanisms remain largely unexplored. A deeper understanding of the genetic basis of *D*_f_ could address these gaps and reveal pathways involved in vascular aging and pave the way for targeted prevention and treatment strategies in aging populations.

We explored the innovative concept of using *D*_f_ as an indicator of vascular aging, enabling the causal biomolecular links between aging and cardiovascular health to be explored. We aimed to uncover pathways involved in aging processes by combining *D*_f_ measures with high-throughput genetic and plasma proteome biomarkers. Specifically, we leveraged genetic factors linked to circulating protein levels to explore causal connections with *D*_f_, as well as genetic factors influencing *D*_f_, to explore causal connections with cardiovascular outcomes and longevity using Mendelian randomization (MR) (*8*).

**Fig. 1.**
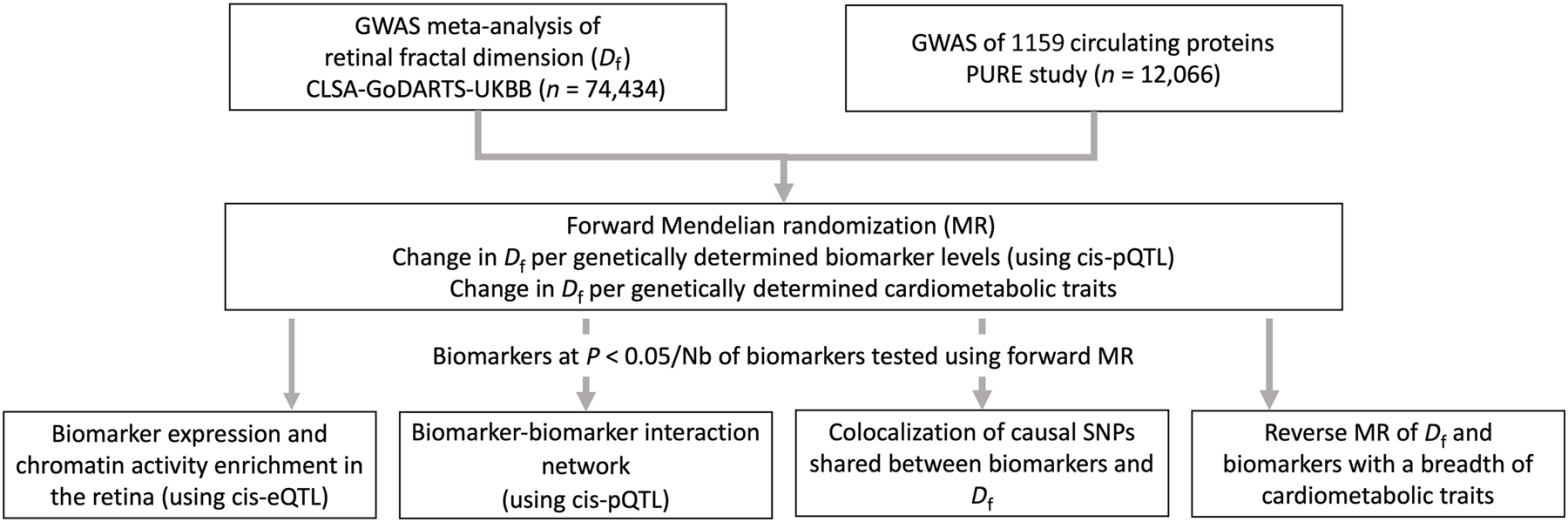
Study design. First, we conducted a large GWAS meta-analysis on retinal *D*_f_ from three cohorts (CLSA, the GoDARTS, and the UKBB). We next performed a proteome-wide MR analysis, which combined genetic determinants of 1159 circulating protein biomarkers from individuals enrolled in the PURE study with *D*_f_. Then, we conducted pathway enrichment analyses, protein interaction network, colocalization, and phenome-wide MR of identified biomarkers to dissect the links between microvascular branching complexity and cardiovascular diseases and longevity. Nb, number.

In our comprehensive multimodal approach, we conducted a large genome-wide association study (GWAS) meta-analysis on retinal *D*_f_ from three expansive epidemiological cohorts, namely, the Canadian Longitudinal Study on Aging (CLSA), the Genetics of Diabetes Audit and Research Tayside Study (GoDARTS), and the UK Biobank (UKBB). We next performed a proteome-wide MR analysis, which combined genetic determinants of 1159 circulating protein biomarkers from individuals enrolled in the Prospective Urban Rural Epidemiological (PURE) study with *D*_f_. This approach allowed us to pinpoint candidate biomarkers for *D*_f_ and investigated whether such biomarkers are also involved in the links between *D*_f_ and cardiovascular diseases and longevity. Then, we conducted pathway enrichment analyses to provide a deeper understanding of the interactions in which the identified biomarkers are involved within the systemic molecular network (Fig. 1). Collectively, our study indicated key pathways and mechanisms involved in microvascular branching complexity, providing insights into potential therapeutic targets for reducing cardiovascular diseases and promoting longevity.

## RESULTS

### GWAS meta-analysis identified one novel locus associated with microvascular branching complexity

**Fig. 2.**
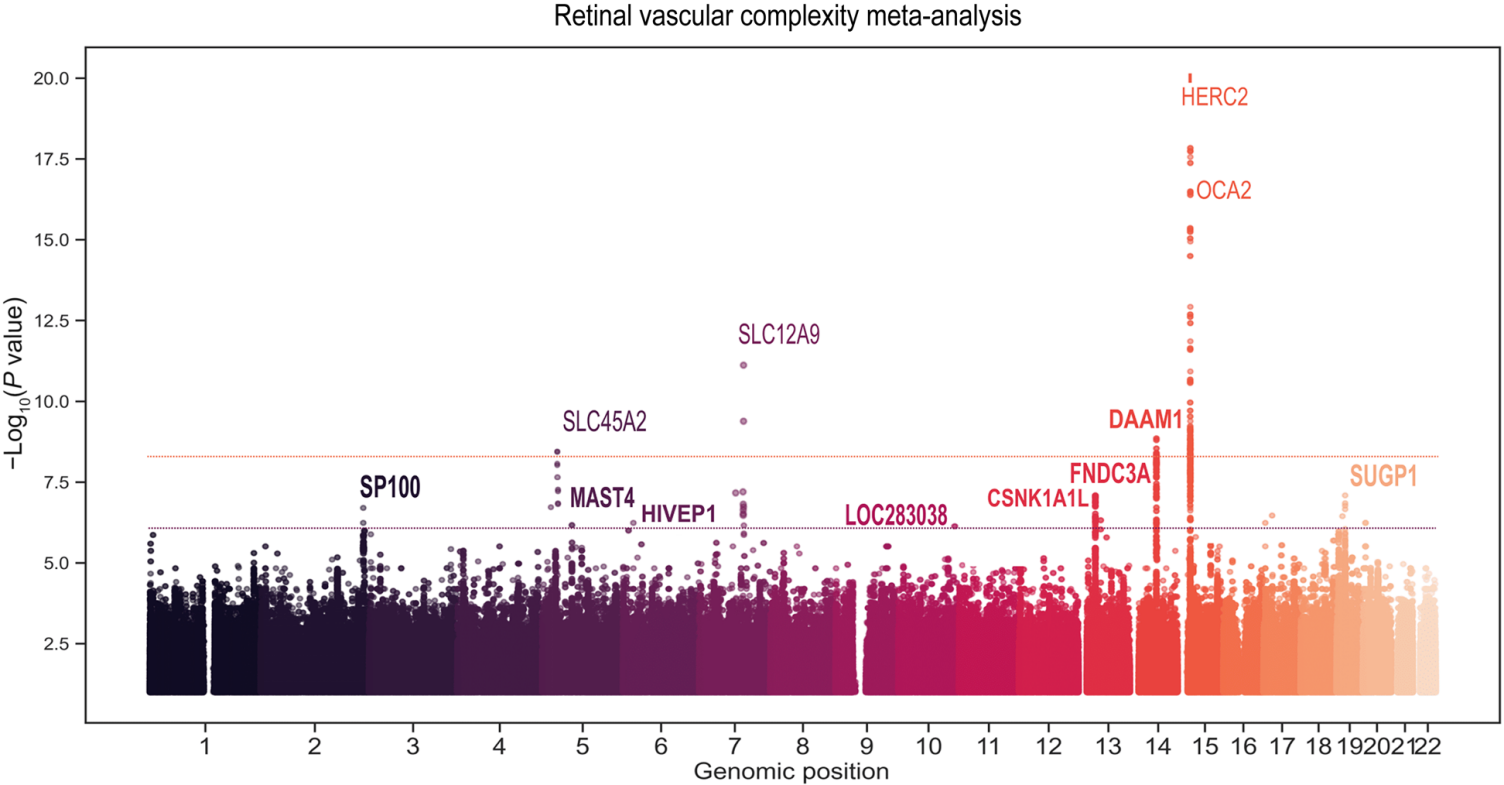
Manhattan plot of the GWAS meta-analysis (CLSA-GoDARTS-UKBB) for *D*_f_. The *x* axis corresponds to the chromosome position, and the *y* axis corresponds to the −log_10_(*P* value) and is truncated at 20 for clarity. Bold gene names indicate significant and suggestive associations.

We performed GWAS meta-analysis for retinal *D*_f_ from 74,434 individuals of European ancestry who participated in CLSA, GoDARTS, or UKBB cohorts. The retinal fundus images available from all individuals in those cohorts were processed by the automatic Vascular Assessment and Measurement Platform for Images of the Retina (VAMPIRE) software to compute *D*_f_, as previously described elsewhere (*9*). The demographic characteristics of each cohort are summarized in tables S1 to S3. First, we conducted the GWAS by fitting a linear model with polygenic effect adjustment in each cohort separately. We then performed an inverse-variance weighted meta-analysis of the GWAS results. The quantile-quantile plot showed an adequate control of genomic inflation λ_GC_ = 1.065 (fig. S1). The meta-analysis revealed five independent genome-wide significant associations (at *P* < 10^−8^) (Fig. 2 and Table 1), including one novel locus and four replicated loci reported in previous GWASs of retinal *D*_f_ (*5*, *10*). This association corresponded to an intronic variant located on chromosome 14 (rs2295848, *P* = 1.41 × 10^−9^) in the *DAAM1* gene, which has been previously related to brain imaging phenotypes (*11*–*14*). Two of the replicated loci were close to *HERC2* (rs12913832, *P* = 2.34 × 10^−59^) and *OCA2* (rs35717941, *P* = 1.20 × 10^−11^), two genes on chromosome 15 that have previously been associated with retinal *D*_f_, pigmentation, and several eye conditions, including cataract, visual acuity, or retinal vessel tortuosity (*14*–*16*). Two other genetic variants near *SLC45A2* on chromosome 5 (5:33956560, *P* = 7.58 × 10^−9^) and *SLC12A9* on chromosome 7 (rs80308281, *P* = 1.51 × 10^−10^) were associated with retinal *D*_f_, both genes being related to hair pigmentation (*17*, *18*). The *SLC12A9* lead variant has also been reported to be associated with arterial blood pressure and resting heart rate (*19*). Additionally, we nominally replicated three genetic variants near *MEFC2*, *IRF4*, and *COLCA1* but did not replicate the genetic variant previously reported near *CTNNB1* (*5*). In addition to these genomic regions, we found seven suggestive associations (at *P* <10^−6^) (table S4). Significant heterogeneity was found between cohorts for five replicated signals, the largest one being for the variants located in the *HERC2/OCA2* gene (heterogeneity, *P* = 5.79 × 10^−96^). Those loci were significantly associated with *D*_f_ in the UKBB and CLSA, but with a stronger effect in the UKBB (fig. S2).

**Table 1.** Results of the GWAS meta-analysis for retinal *D*_f_ from the CLSA-GoDARTS-UKBB cohorts. SNP, single-nucleotide polymorphism; CHR, chromosome; POS, position; FREQ, effect allele frequency.

SNP	CHR	POS	GENE	Effect allele	FREQ	Meta-analysis				Previously published
						Effect estimate	SD	−Log_10_(*P* value)	Heterogeneity *P* value	
rs2295848	14	59781050	*DAAM1*	T	0.18	−0.0009	1 × 10^−4^	8.85	0.18	Novel association
rs16339	3	41281600	*CTNNB1*	G	0.55	−0.0002	1 × 10^−4^	1.1	1.0	Reported in (*5*)
5:33956560_GT_G	5	33956560	*SLC45A2*	G	0.032	−0.0040	7 × 10^−4^	8.12	1.0	Reported in (*5*, *17*, *18*)
rs17421410	5	87836307	*MEF2C*	G	0.926	0.0006	2 × 10^−4^	2.78	0.0009	Reported in (*5*)
rs12203592	6	396321	*IRF4*	T	0.79	0.0005	1 × 10^−4^	4.32	8.6 × 10^−11^	Reported in (*5*)
rs80308281	7	100457578	*SLC12A9-GNB2*	T	0.005	0.0085	1 × 10^−3^	9.82	0.004	Reported in (*5*, *17*–*19*)
rs10502124	11	110894702	*COLCA1*	A	0.37	−0.0004	1 × 10^−4^	3.07	0.0003	Reported in (*5*)
rs12913832	15	28365618	*HERC2*	A	0.22	0.0022	1 × 10^−4^	58.93	5.8 × 10^−96^	Reported in (*5*, *14*–*16*)
rs35717941	15	28286405	*OCA2*	A	0.90	−0.0023	3 × 10^−4^	10.92	1.0	Reported in (*5*, *14*–*16*)

Subsequent gene-level analysis of GWAS using the multimarker analysis of genomic annotation (MAGMA) tool (*20*) showed an enrichment of notable genes in pathways related to melanin biosynthesis and inflammatory response (tables S5 and S6). Last, we estimated the genetic correlations of *D*_f_ with systemic inflammation and cardiometabolic outcomes. We found nominally significant negative correlations of *D*_f_ with C-reactive protein (*r*_g_ = −0.13 ± 0.05), atherosclerosis (*r*_g_ = −0.24 ± 0.03), coronary artery disease (*r*_g_ = −0.12 ± 0.06), stroke (*r*_g_ = −0.23 ± 0.09), and a positive correlation with longevity (*r*_g_ = 0.12 ± 0.06) (table S7). Such findings were consistent with the findings that we previously reported for analysis conducted in a subset of UKBB individuals (*10*).

### MR and colocalization demonstrate causal associations for immune and inflammatory biomarkers with microvascular branching complexity

**Fig. 3.**
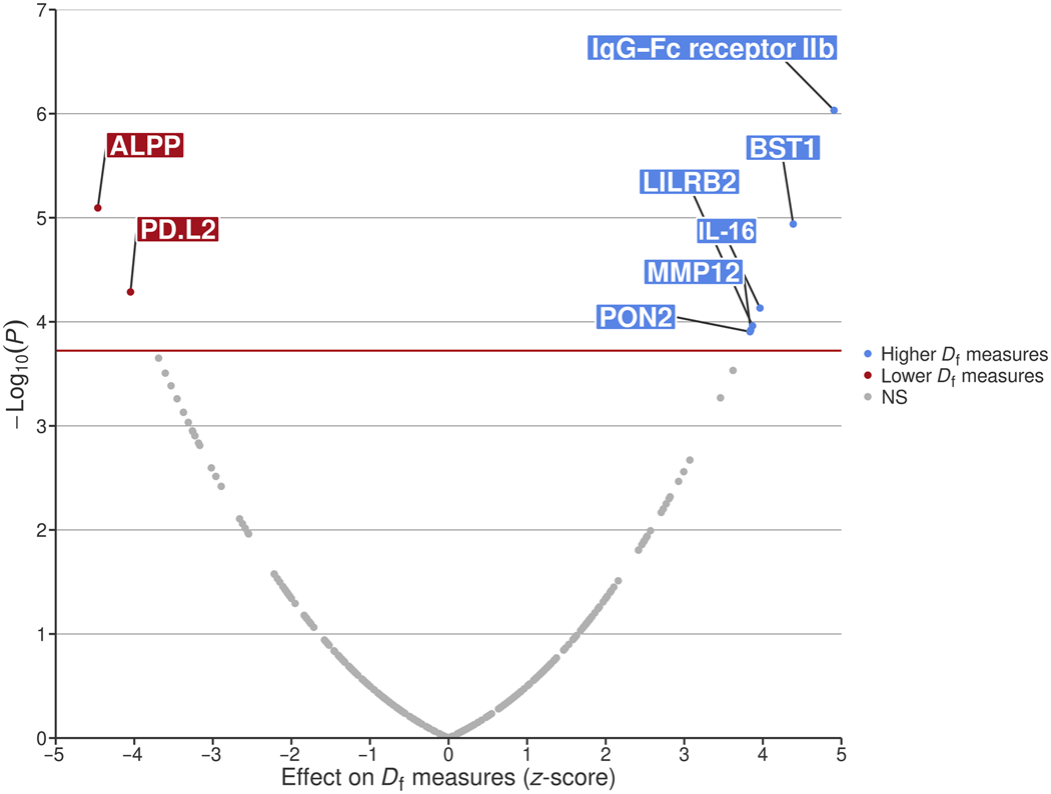
Volcano plot of circulating biomarkers associated with *D*_f_, identified by MR. The *x* axis corresponds to the effect of each biomarker on *D*_f_ measure (given in *z*-score values); the *y* axis corresponds to the −log_10_(*P* value), obtained using inverse variance weighted (IVW) MR method. The horizontal red line corresponds to the Bonferroni significant *P* value threshold. ALPP, alkaline phosphatase, placental type; PDL2, programmed cell death 1 ligand 2; IgG–Fc RecIIb, immunoglobulin G–Fc receptor IIb; BST1, bone marrow stromal antigen 1; LILRB2, leukocyte immunoglobulin-like receptor subfamily B member 2; IL-16, pro–interleukin-16; MMP12, matrix metalloproteinase-12; PON2, serum paraoxonase/arylesterase 2; NS, not significant.

We screened a panel of 1159 plasma biomarkers involved in inflammation and cardiometabolic pathways (*21*) for putative causal associations with *D*_f_ using a two-sample bidirectional MR analysis. A subset of 264 biomarkers had suitable genetic instruments to be retained in the forward MR, including circulating biomarkers as exposures and retinal *D*_f_ as outcome. We found evidence of an association between genetically predicted biomarker concentrations and *D*_f_ for eight of those biomarkers beyond the Bonferroni-corrected significance threshold [at inverse variance weighted (IVW), *P* < 0.05/264 = 1.89 × 10^−4^]. Genetically determined circulating levels of immunoglobulin G (IgG)–Fc receptor IIb (RecIIb), bone marrow stromal antigen 1 (BST1), leukocyte immunoglobulin-like receptor subfamily B member 2 (LILRB2), interleukin-16 (IL-16), matrix metalloproteinase-12 (MMP12), and serum paraoxonase/arylesterase 2 (PON2) were positively associated with *D*_f_, while genetically determined circulating levels of alkaline phosphatase, placental type (ALPP) and programmed cell death 1 ligand 2 (PDL2) were negatively associated with *D*_f_ (Fig. 3 and table S8). We next performed stratified analyses according to diabetes status due to the higher representation of individuals with diabetes in our GWAS meta-analysis. Associations for LILRB2, MMP12, PON2, and ALPP were consistent, while additional positive associations for TCN2 levels and negative associations for matrilin-3, transmembrane protease serine 5, and netrin receptor UNC5C with *D*_f_ were observed in individuals without diabetes (*P* < 0.05/136 = 3.68 × 10^−4^) (table S9). Positive associations of Ficolin 2, Serpin family A member 9, Dopa decarboxylase, IL-2RA, and Legumain and negative associations of Complement C1q A chain, Neural cell adhesion molecule 1, and Cytotoxic and regulatory T-cell molecule levels were also observed with *D*_f_ in individuals with diabetes (*P* < 0.05/244 = 2.05 × 10^−4^) (table S10). A reverse MR analysis, using *D*_f_ as exposure and a total of 1159 unique biomarkers as outcomes did not reveal significant associations (*P* < 0.05/1159 = 4.3 × 10^−5^) and ruled out a reverse causation of *D*_f_ on circulating biomarker levels (table S11).

### Phenome-wide MR revealed the link between biomarkers, cardiovascular diseases and longevity, and microvascular branching complexity

**Fig. 4.**
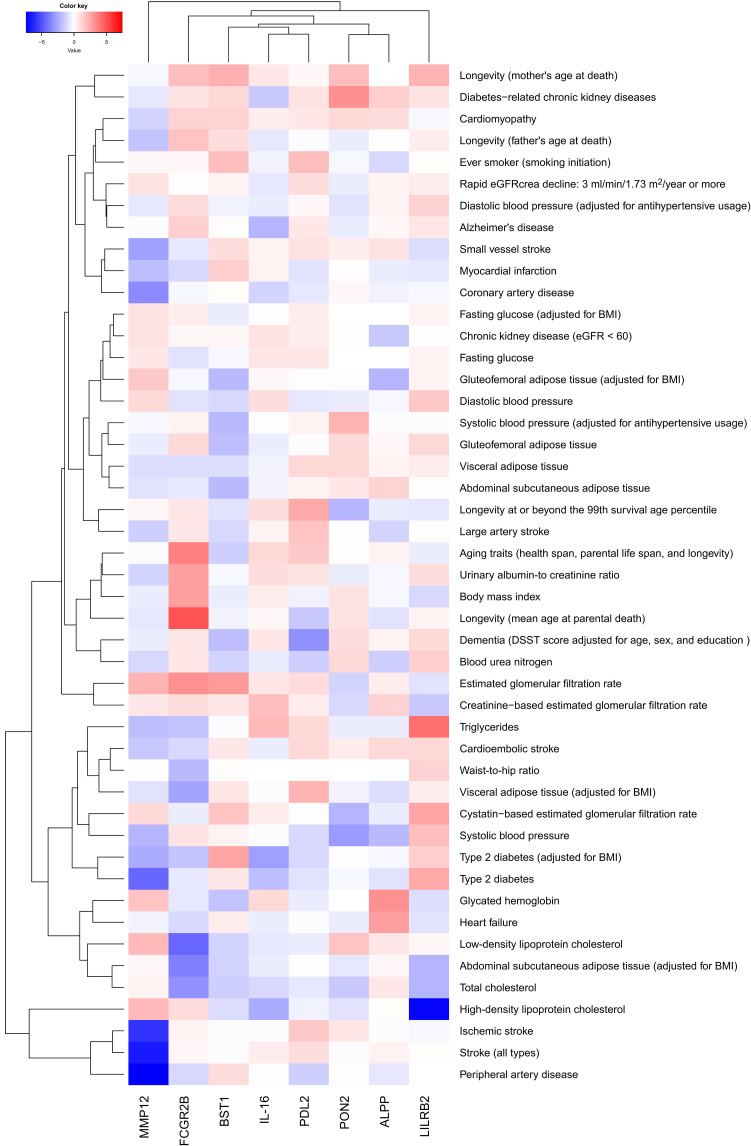
Heatmap of the associations between significant biomarkers for *D*_f_ and cardiometabolic outcomes. Hierarchical heatmap represents the effects (*z*-score) of each of the eight biomarkers related to *D*_f_ on each of the 47 cardiometabolic outcomes. Red color corresponds to positive associations between genetically determined biomarker levels and outcomes, and blue color corresponds to negative associations; darker colors correspond to stronger associations. Dendrograms reflect the distance (or similarity) in the associations between columns (biomarker levels) and rows (outcomes). eGFRcrea, estimated Glomerular Filtration Rate from creatinine. DSST, Digit Symbol Substitution Test.

We conducted a phenome-wide MR to investigate the associations of retinal *D*_f_ and biomarkers related to *D*_f_, with a broad range of cardiometabolic outcomes, encompassing 47 distinct phenotypes for which the GWAS summary statistics were extracted from publicly available consortium data (listed in table S12). First, among the eight *D*_f_-related biomarkers, three were also significantly associated with cardiometabolic disease phenotypes (*P* < 0.05/(8 × 47) = 1.33 × 10^−4^). Higher genetically predicted IgG–Fc RecIIb levels were associated with longer parental life span [mean change of 0.02 (±0.003) year per 1 SD increase in biomarker level; *P* = 5.5 × 10^−7^] and decreased low-density lipoprotein–cholesterol levels [mean change of −0.01 (±0.002) mM per 1 SD increase in biomarker level; *P* = 1.24 × 10^−5^]. Higher MMP12 levels were associated with a lower risk of stroke [odds ratio (OR) per 1 SD increase in biomarker level, 0.92; 95% confidence interval (CI), 0.90 to 0.95; *P* = 5.96 × 10^−11^] and, in particular, with stroke of ischemic cause (OR, 0.91; 95% CI, 0.89 to 0.94); *P* = 6.90 × 10^−9^), as well as with a lower risk of peripheral artery disease (OR, 0.86; 95% CI, 0.83 to 0.90; *P* = 1.55 × 10^−13^), T2D (OR, 0.95; 95% CI, 0.93 to 0.97; *P* = 1.65 × 10^−5^), and coronary artery disease (OR, 0.92; 95% CI, 0.87 to 0.96; *P* = 1.28 × 10^−4^). Higher LILRB2 levels were associated with lower high-density lipoprotein–cholesterol levels (mean change of −0.06, ±0.008 mM; *P* = 6.43 × 10^−13^) and higher triglycerides levels (0.02, ±0.004 mM; *P* = 3.14 × 10^−7^) (Fig. 4 and table S13). Next, phenome-wide MR of cardiometabolic diseases on *D*_f_ showed significant positive relationships with Body mass index (increase by 8.77 × 10^−3^ in *D*_f_ per 1 unit increase BMI (±2.19 × 10^−3^; *P* = 6.05 × 10^−5^), systolic blood pressure (increase by 5.98 × 10^−5^ (±1.20 × 10^−5^; *P* = 6.48 × 10^−7^), and systolic blood pressure adjusted for antihypertensive medication usage (increase by 5.52 × 10^−5^, ±1.22 × 10^−5^; *P* = 6.00 × 10^−6^) (all *P* values < 0.05/47 = 1.06 × 10^−3^) (table S14). A reverse phenome-wide MR of *D*_f_ on cardiometabolic diseases only showed significant inverse relationships with estimated glomerular filtration rate (*P* < 0.05/47 = 1.06 × 10^−3^) (table S15).

Multivariate MR investigating the effects of the identified biomarkers on diseases, adjusted for their effects on *D*_f_, confirmed that the effects of IgG–Fc RecIIb on longevity were partially mediated by an effect on *D*_f_, with an attenuation of the effect of IgG–Fc RecIIb on longevity by 34% when adjusting on *D*_f_ [*D*_f_ adjusted mean change of 0.01 (±0.002) year per 1 SD increase in biomarker level; *P* = 3.31 × 10^−12^]. The effects of MMP12 levels on cardiovascular outcomes were also partially mediated by its effects on *D*_f_, but to a lesser extent (ranging from 9 to 15% of effect attenuation after adjustment on *D*_f_) (Table 2).

**Table 2.** MR results of biomarkers associated with cardiovascular disease and longevity, adjusted for their effects on retinal *D*_f_. IVW, inverse variance weighted; LCI, lower confidence interval; UCI, upper confidence interval; Prop., proportion; Nb snps, number of snaps.

Biomarker	Outcome	Nb snps	MR method	Effect estimate (per 1 SD biomarker level)	SE	*P* value	Odds ratio (per 1 SD biomarker level)	95% LCI	95% UCI	Prop. of mediation
IgG–Fc receptor IIb	Parental life span (years)	16	IVW	0.0166	0.0033	5.41 × 10^−7^				0.33
			Multivariate IVW	0.0112	0.0018	1.90 × 10^−10^				
MMP12	Stroke (all types)	9	IVW	−0.0807	0.0123	5.96 × 10^−11^	0.92	0.90	0.94	0.09
			Multivariate IVW	−0.0737	0.0068	2.04 × 10^−27^	0.93	0.92	0.94	
	Ischemic stroke	9	IVW	−0.0902	0.0156	6.90 × 10^−9^	0.91	0.89	0.94	0.12
			Multivariate IVW	−0.0796	0.0073	1.80 × 10^−27^	0.92	0.91	0.94	
	Peripheral artery disease	9	IVW	−0.1498	0.0203	1.55 × 10^−13^	0.86	0.83	0.90	0.15
			Multivariate IVW	−0.1272	0.0105	4.30 × 10^−34^	0.88	0.86	0.90	
	Coronary artery disease	9	IVW	−0.0794	0.0207	1.28 × 10^−4^	0.92	0.89	0.96	0.12
			Multivariate IVW	−0.0696	0.0115	1.32 × 10^−9^	0.93	0.91	0.95	

**Fig. 5.**
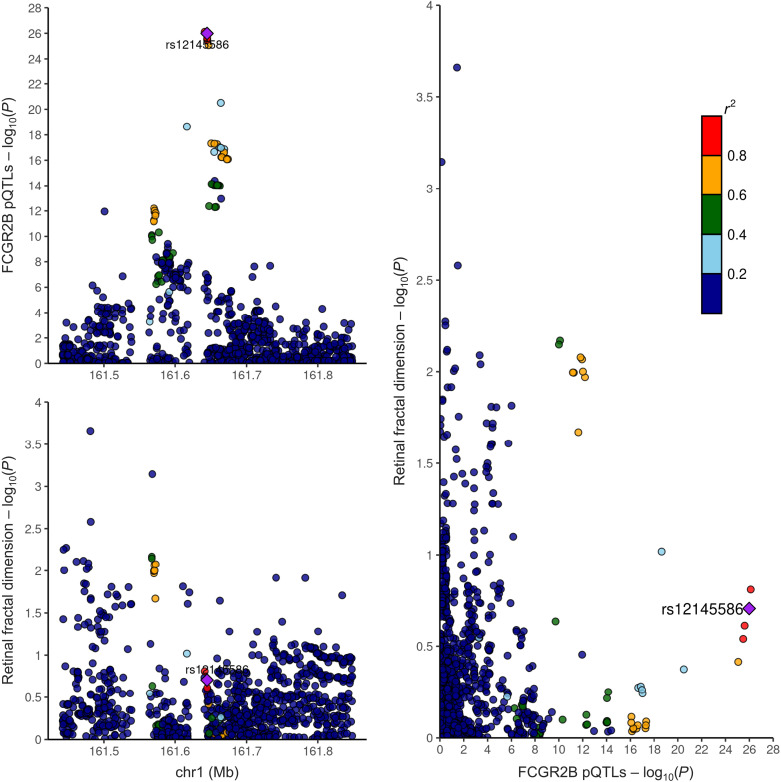
Colocalization of *FCGR2B* with *D*_f_. Top left: Plots represent the associations of genetic variants with *D*_f_ at the locus 1: 161.4 to 161.9 Mb, with the *x* axis being the chromosome location and the *y* axis being the log_10_ *P* value of the associations. Bottom left: Plots represent the associations of genetic variants with circulating IgG–Fc RecIIb levels (encoded by *FCGR2B*) at the locus 1: 161.4 to 161.9 Mb, with the *x* axis being the chromosome location and the *y* axis being the log_10_ *P* value of the associations. Right: Plots represent the genetic correlation (*r*^2^) of circulating IgG–Fc RecIIb levels with *D*_f_ at the locus 1: 161.4 to 161.9 Mb, with darker colors showing stronger associations.

Moreover, colocalization analysis confirmed that circulating IgG–Fc RecIIb levels (encoded by *FCGR2B* gene) and retinal *D*_f_ shared a common causal genetic variant. The effect allele C of the variant rs12145586-C/T was associated with a decrease by −1.08 SD in circulating IgG–Fc RecIIb level (±0.03 SD; *P* = 1.48 × 10^−262^) and a decrease by −2.10 × 10^−4^ in *D*_f_ (±1.6 × 10^−4^; *P* = 1.97 × 10^−1^), with a posterior probability superior to 80% (i.e., H3 + H4 ≥ 0.8) (Fig. 5 and table S16). To investigate which cells might drive this *FCGR2B* protein quantitative trait locus (pQTL) association, we conducted further colocalization analyses between *FCGR2B* pQTL and single-cell expression quantitative trait loci (sc-eQTL) in peripheral blood mononuclear cells (PMBCs) (*22*). This supported the presence of the causal variant effect in *FCGR2B* across different cell types, and most of which were involved in the immune response (table S17 and fig. S3)

### Network analysis of biomarker-related to microvascular branching complexity confirms enrichment for immune and inflammatory pathways

**Fig. 6.**
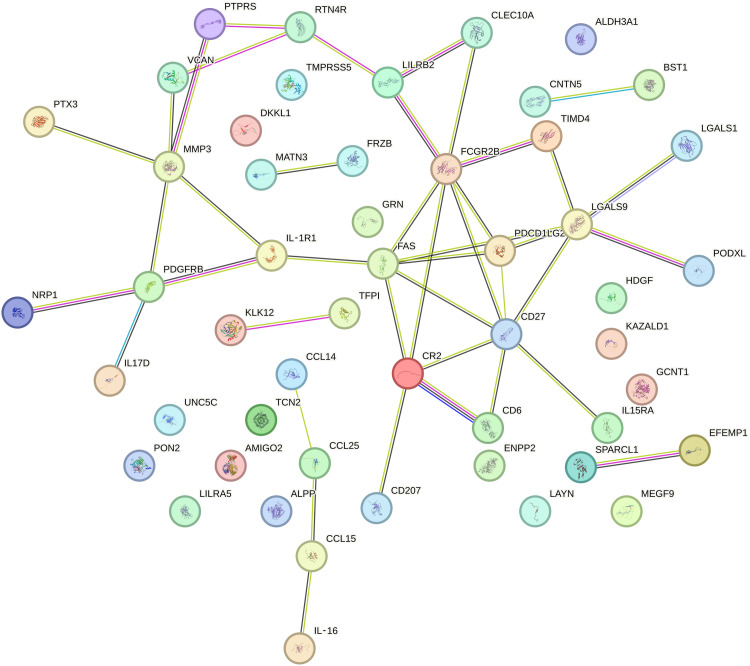
Protein interaction network of biomarkers associated with *D*_f_. Figure has been generated using STRING (*88*) in which significant biomarkers for *D*_f_ (MR association, *P* values of <0.001) were used as inputs. Network nodes represent proteins, and colored nodes are query proteins (in red) and first shell of interactors. Edges represent protein-protein associations.

We reinforced the MR findings with protein-protein interaction networks, which revealed significant enrichment for pathways involved in immune and inflammatory responses, such as cytokine-cytokine receptor interaction pathway [false discovery rate (FDR) = 4.12 × 10^−5^] and immune-related disease (FDR = 0.015) (Fig. 6 and tables S18 and S19). We also investigated the expression levels and chromatin activity of the *D*_f_ -associated biomarkers in the retinal tissue. Of the eight MR significant biomarkers, three (IgG–Fc RecIIb, PON2, and IL-16) were expressed in retinal tissue and associated with open chromatin regions and related to immune and metabolic networks (table S20) (*23*).

## DISCUSSION

Using the largest GWAS meta-analysis for retinal *D*_f_ to date as inputs, we identified one novel locus associated with *D*_f_ and replicated the associations of seven previously identified variants (*5*, *10*). We also confirmed genetic correlations between *D*_f_ and systemic inflammation, cardiovascular disease, stroke, and longevity and identified circulating MMP12 and IgG–Fc RecIIb levels as key mediators linking *D*_f_ to cardiovascular and longevity outcomes. Notably, enrichment analyses also highlighted the role of IgG–Fc RecIIb in immune and inflammatory responses to aging, processes referred to as inflammaging.

The findings from the *D*_f_ GWAS meta-analysis were consistent with previous genetic analyses on retinal *D*_f_, thus supporting the biologically coherent relationships of *D*_f_ -associated variants with various ocular and cardiovascular traits and diseases (*5*, *10*, *24*). We found that several loci were also associated with either cardiovascular risk factors (e.g., hypertension and dyslipidemia) or diseases (e.g., coronary artery diseases), as well as inflammatory and immune responses (*25*–*33*).

Previous studies focused on the links between *D*_f_ and diseases and showed that a lower *D*_f_ was associated with a higher risk of mortality, incident hypertension, congestive heart failure, renal failure, T2D, sleep apnea, anemia, and multiple ocular conditions and was causally associated with skin cancer and retinal detachment (*5*, *10*). However, none of those studies investigated the molecular mechanisms linking *D*_f_ to such diseases. Our study is the first MR analysis investigating the relationships between the proteome and *D*_f_ and provides evidence on the role of eight circulating biomarkers in microvascular changes, including IgG–Fc RecIIb, BST1, LILRB2, IL-16, MMP12, PON2, ALPP, and PDL2. Additionally, we investigated the associations between those eight biomarkers and health-related traits. Notably, IgG–Fc RecIIb and MMP12 also emerged as causal mediators of diseases, linking *D*_f_ to longevity and to various chronic diseases, including stroke, peripheral artery disease, and T2D, respectively. The IgG–Fc RecIIb effect size on life span was in the range of previously reported biomarkers for longevity (*34*). The MR findings were reinforced by single-cell colocalization analysis in PMBCs, which confirmed the putative causal role of IgG–Fc RecIIb on *D*_f_ changes driven by its expression in immune cells, and by enrichment analysis that indicated that IgG–Fc RecIIb expression was associated with different regions of the retinal regulatory network.

Biologically, IgG–Fc RecIIb is a densely expressed receptor that plays a role in the immune response and antiviral activity of malt cells, basophils, eosinophils, macrophages, monocytes, dendritic cells, and B cells in the spleen and lymph nodes (*35*). Previous studies showed that variants leading to impaired FcγRecIIb activity cause the accumulation of IgGs, B cell apoptosis, and proinflammatory states (*36*, *37*). Efforts to pharmacologically modulate FcγRecIIb either directly by regulating immune responses or indirectly by influencing FcγRecIIb interactions through other pathways, using monoclonal antibodies and protein fusion approaches, have produced drugs, such as obinutuzumab or rituximab with applications in immune diseases and hematologic cancers (*38*, *39*).

The MR findings were consistent with previous plasma proteome studies that showed that circulating MMP12 levels were linked to cardiovascular outcomes, including heart disease, peripheral artery disease, and stroke (*40*–*43*). Higher genetically predicted levels of MMP12 have consistently been associated with a lower risk of stroke, and it has been suggested as potential therapeutic target (*44*). Biologically, MMP12 belongs to a family of endopeptidases that degrade structural components of the extracellular matrix (*45*). MMP12 plays key pro- and anti-inflammatory roles in the body through the cleavage of cytokines and subsequent regulation of cell migration and signaling and may contribute to atherosclerotic cardiovascular disease pathogenesis through the regulation of macrophage migration, plaque development, and rupture (*45*).

Chronic inflammation related to aging has been characterized as a clinical condition, named inflammaging, associated with a high risk of prematurely developing age-related diseases and adverse health outcomes (*46*, *47*). This observation is consistent with our complementary biomarker analysis that revealed associations between IgG–Fc RecIIb and life span, and MMP12 levels and cardiovascular diseases. Considering that genetic predictions of IgG–Fc RecIIb and MMP12 levels are also associated with *D*_f_, we propose *D*_f_ as a potential accessible imaging marker of the inflammatory status related to aging, such that a lower *D*_f_ would be indicative of a higher inflammaging status.

Although this was the largest genetic study on retinal vascular traits to date, to validate our results and extrapolate them to a valid clinical outcome, it is of utmost importance that studies include individuals of non-European ancestry. Although we measured 1159 selected circulating proteins, this still represents a small subset of the ~20,000 proteins in the human proteome (*48*). From a methodological perspective, MR relies on several assumptions that cannot all be assessed, although our analyses generally showed concordant effect estimates between different MR approaches. For instance, when assessing the significance of the Egger intercept test, we used a conservative approach of not correcting for multiple testing; however, our power to detect directional pleiotropy may have been insufficient (*49*). If undetected directional pleiotropy did not affect the results, then the lack of concordance between the MR-Egger estimates and the other MR estimates might be attributable to a violation of an assumption of MR-Egger (*50*). We may also have limited power to assess colocalization between variants associated with protein level and *D*_f_, leading to the possibility that significant MR findings might reflect the presence of separate causal variants in linkage disequilibrium (LD) with one another (*51*). We measured protein expression levels in plasma, but not in the retina. However, the use of cis*-*pQTLs, which are likely to be shared across tissues (*52*), as instrumental variables in these MR analyses, supports the possibility that the MR associations reflect the actions of the proteins of interest in the retina. Additionally, there are little publicly available data for the retinal transcriptome and proteome to perform further enrichment analysis. This lack of available information and rarity of studies is driven by the invasiveness of collecting retinal tissue and for the complex extraction of proteins and DNA from such samples.

In conclusion, our study identified IgG–Fc RecIIb and MMP12 as key mediators in immune and inflammation pathways, linking lower microvascular branching complexity to a higher risk of cardiovascular diseases and a shorter life span. Therefore, retinal *D*_f_ may be a convenient marker to estimate inflammaging, such that lower retinal *D*_f_ may indicate a higher inflammaging status. Last, these findings pave the way for strategies targeting IgG–Fc RecIIb and MMP12 to promote longevity by mitigating age-related immune and inflammatory pathways involved in cardiovascular diseases.

## MATERIALS AND METHODS

### Study populations

The UKBB (www.ukbiobank.ac.uk) is a large multisite cohort study that consists of 502,655 individuals aged between 40 and 69 years at baseline and recruited from 22 centers across the UK between 2006 and 2010. The study was approved by the National Research Ethics Committee, reference 11/NW/0382, and informed consent was obtained from all participants as part of the recruitment and assessment process. From these, a baseline questionnaire, physical measurements, and biological samples were undertaken for each participant. Ophthalmic examination was not included in the original baseline assessment and was introduced as an enhancement in six UKBB centers across the UK. This examination consisted in capturing paired retinal fundus with a 45° primary field of view obtained using a Topcon 3D OCT-1000 MKII (Topcon Corporation). These analyses were completed using fundus images collected at the first and the second ophthalmic examination visits from 49,712 participants of white European ancestry with at least one eye image of good quality. The data were processed as published in our previous analysis (*10*). The study was approved by the National Research Ethics Committee, reference 11/NW/0382, and informed consent was obtained from all participants as part of the recruitment and assessment process.

Participants from Genetics of Diabetes Audit and Research in Tayside Scotland (GoDARTS) (*53*) and Genetics of Scottish Health Research Register (GoSHARE) (*54*) were used for GWAS analysis. GoDARTS is a cohort study in the Tayside region of Scotland that began recruiting participants in 1996 and continued to 2015. The study included 10,149 individuals with T2D and 8157 control subjects without T2D at the time of recruitment. GoSHARE data on clinical and lifestyle parameters were collected at the time of recruitment, and participants also provided consent to their electronic health record linkage. Retinal photographs used for the Scottish national diabetes retina screening (DRS) are available for all patients with diabetes in GoDARTS (*55*). DRS photographs were obtained using a standardized protocol with a 45° view centered on the macula. Participants also provided a sample of blood for genome-wide genotyping that was performed using multiple separate genotyping arrays, including the Affymetrix version 6.0, Illumina OmniExpress BeadChips, Illumina Infinium Broad BeadChips, Illumina Human OmniExpressExome-8 version 1.0 BeadChip, and Illumina OmniExpressExome-8 version 1.2 BeadChip. Genetic data were available for 7722 T2D participants after quality control. GWAS data were then obtained using the Affymetrix Genome-Wide Human SNP Array 6.0 the Illumina HumanOmniExpress and Broad. The Affymetrix GWAS chip contains 932,979 single-nucleotide polymorphisms (SNPs), and the Illumina GWAS chip contains 731,296 SNPs. Then, imputation of additional and missing genotypes were performed by SHAPEIT (*56*) and IMPUTE2 (*57*) using the 1000 Genomes reference panel (*58*). In addition, 707 T2D cases and 3478 controls were genotyped using custom genotyping arrays from Illumina. Such arrays include the Immunochip, Cardio-Metabochip (Metabochip), and Human Exome array. The Immunochip contains 196,524 genetic markers from loci that have previously been associated with at least 1 of the 13 autoimmune diseases, including type 1 diabetes (*59*), while the Metabochip contains 196,725 SNPs from loci that have prior evidence of associations with T2D, coronary artery disease/myocardial infarction, and 21 related traits (*60*). The Human Exome Array contains 247,870 genetic markers from across the exome, allowing for studies to focus on identifying protein-altering variants (*61*). The genotyping process for the GoDARTS and GoSHARE cohorts involved various platforms, including Affymetrix 6.0, Illumina Omni Express-12VI, and GSA v2.0. After applying quality control criteria, a total of 7722 participants (6249 from GoDARTS and 1473 from GoSHARE) were considered for analysis. The quality control criteria used to exclude individuals from analysis were individual genotype call rate of <95%, discrepancy in gender, heterozygosity of >3 SDs from the mean, and highly related samples identified through identity by descent analysis. SNP-level quality control was performed by excluding markers with a call rate of less than 95% and those with a Hardy-Weinberg *P* <1 × 10^−6^. PLINK versions 1.7 and 1.9 were used for quality control assessment and data preprocessing for imputation. Ancestry outliers were detected using principal components analysis in each cohort. The genotype data from all three cohorts were then imputed against the HRC r1.1 reference panel. Monomorphic markers and those with an imputation quality score below 0.4 were excluded from the postimputation data. The GoDARTS study has been approved by the East of Scotland Research Ethics Committee (Dundee, UK).

The CLSA is a large, national, stratified, random sample of 50,000 Canadians aged 45 to 85 years at the time of recruitment (2010 to 2015) and followed until 2033 (or until death). The CLSA aims to investigate how various factors (e.g., biological, physical, psychological, social, and environmental factors), individually and in combination, influence the health and well-being of aging individuals (*62*). A subset of 30,097 participants (i.e., comprehensive cohort) had physical examinations and biological specimen collection, including fundus photographs (one for each eye) obtained using the Topcon TRC-NW8 nonmydriatic retinal camera. A total of 50,957 retinal photographs from 25,717 CLSA participants, were analyzed using VAMPIRE (version 3.1, University of Edinburgh and University of Dundee, UK) to compute image quality (good/moderate/poor) and the *D*_f_ of the retinal vascular pattern. Participants with poor image quality for both eyes were excluded from subsequent analyses. Among the comprehensive subset, 26,622 CLSA participants (with 93% of Europeans) were successfully genotyped using the UKBB Array (*63*). The quality control steps have been detailed elsewhere (*64*). Briefly, phasing and imputation were conducted using the TOPMed reference panel at the University of Michigan Imputation Service. We used the TOPMed reference panel version r2 and then prephased and imputed the genotype data using EAGLE2 and Minimac, respectively, for both autosomal and X chromosomes. Samples with low call rates (<95%), sex mismatches, or cryptic relatedness were removed. Imputed SNPs were excluded on the basis of low call rates (<95%), deviation from Hardy-Weinberg (*P* < 10^–6^), low minor allele frequency (MAF; <0.0001), and low imputation quality (Rsq < 0.6). Research ethics approval was granted by the Hamilton Integrated Research Ethics Board (HiREB, no. 7764).

The Prospective Urban Rural Epidemiology (PURE) study is a large prospective study of individuals in 27 low-income, middle-income, and high-income countries. Participant recruitment and selection has been described in detail elsewhere (*65*). Briefly, the PURE study collected socio-demographics, physical examination, routine laboratory results (e.g., lipid profile), lifestyles, depression and stress questionnaires, disease status and age at diagnosis, medication usage, and family health history at recruitment and each follow-up visit. A biobanking initiative was developed for a subset of PURE participants recruited between 5 January 2005 and 31 December 2006 to assess genomic and proteomic markers of chronic disease risk, based on a case-cohort design. Blood samples from participants were transported from 14 countries (i.e., Argentina, Bangladesh, Brazil, Canada, Chile, Colombia, Iran, Pakistan, Philippines, South Africa, Sweden, Tanzania, United Arab Emirates, and Zimbabwe) to the Population Health Research Institute (Hamilton, ON, Canada) and stored at −165°C. Samples were considered eligible if they belonged to individuals from the major self-reported ethnicity in the residing country (e.g., European ancestry in Sweden). Samples were deemed ineligible if they were unsuitable for analysis or were non-fasting. Hereby, we selected a random sample from the pool of 55,246 eligible participants and then included all individuals who had incident events of interest that were not selected as part of the random sample. The final sample set included 12,066 PURE participants. The research ethics committees at each study location, including Hamilton Health Sciences, approved the project (HiREB, no. 8089).

### Fractal dimension

Retinal fractal dimension (commonly abbreviated as *D*_f_) is a measure of vasculature density and branching complexity, as previously described elsewhere (*66*). *D*_f_ measurements were obtained after fundus image segmentation with VAMPIRE V3.1 software for each cohort, using validated methods for image preprocessing, vessel segmentation map, and *D*_f_ estimation (*9*, *67*, *68*). The three datasets included participants with both eyes analyzed. In the cases of CLSA and UKB, if both images were of a similar good quality, the mean *D*_f_ was used; otherwise, the image with the highest quality was chosen. For GoDARTS, we included the mean *D*_f_ if both images were available.

### Fundus image quality

Fundus image quality measurement in CLSA was defined through manual evaluation of the vessel segmentation map generated by the VAMPIRE V3.1 software. As for the UKB, this quality evaluation, named image quality score (IQS), was derived from a prior study (*69*). The software perform automatically detects the retinal vasculature, creating a binary vessel map for each image, thus, following the pipeline described in (*10*). Images with IQS > 0 were classified as good quality and were included in the pipeline. When participants had paired good quality images, the mean *D*_f_ and the mean IQS were used for subsequent analysis in the CLSA and UKB datasets. GoDARTs did not include image quality measurements, but retinal images were subject to a manual quality control step during the ophthalmic examination to ensure suitability for phenotype.

### GWAS meta-analysis for *D*_f_

We established a pipeline to complete the same GWAS across each cohort. The GWAS model was an additive mixed linear model that adjusted for age, sex, fundus IQS, and the first 10 genetic principal components. Because GoDARTS is a cohort to study diabetes, the GoDARTS GWAS was also adjusted for the presence of diabetic retinopathy. This process was completed using REGENIE software (*70*), using genotyped SNP data for the first step, and imputed SNP information for the second step.

Variants included for the meta-analysis were selected independently in each cohort. Variants were autosomal SNPs present in the genotyping and imputing panel with a Hardy-Weinberg equilibrium (HWE) >10^−6^, MAF > 5 × 10^−3^, call rate > 0.9, and imputation score > 0.8. The number of total SNPs analyzed after quality control was 30,275,286 SNPs in the UKB; 11,950,600 SNPs in the CLSA; and 8,059,472 SNPs in the GoDARTS. The meta-analysis was completed using a fixed effect inverse variance–based model using METAL software (*71*). Additionally, we completed a random-effects inverse variance meta-analysis in the significantly heterogenic genetic variants from the fixed-effects meta-analysis using the meta package in R (*72*). We visualized the effect of SNPs across cohorts using forest plots in the meta package in R (*72*). Loci in the meta-analysis were defined using the clump function of PLINK1.9 and UKBB European ancestry genotypes as the LD reference panel. Clumping parameters were genetic correlation (*r*^2^) = 0.1, *P* = 1 × 10^−6^, *P*2 = 0.01, and kilobase = 10,000 base pairs. Associated genes for independent loci were established using the nearest gene strategy. Gene level analyses were conducted by MAGMA software (*20*, *73*).

### Genetic correlation of *D*_f_ with systemic inflammation and cardiometabolic outcomes

Genomic inflation and genome-wide genetic correlation estimates (*r*_g_) for completed GWASs were estimated using the LD score and High-Density Lipoprotein (HDL) cholesterol HDL software (*74*, *75*).

### Phenome-wide association studies

Phenome-wide association analysis was completed to investigate the effects of significant SNPs from the meta-analysis on other traits. To this end, independent lead SNPs were searched in GWAS Catalog (*76*) and GeneATLAS (*77*), databases that contain numerous GWAS summary statistics for diverse traits and diseases. Genetic variants must have a *P* value of less than 1 × 10^−8^ on the trait to assume a significant association.

### Circulating protein biomarker measurement

A 1.8-ml aliquot of plasma from each PURE participant was processed in the Clinical Research Laboratory and Biobank in Hamilton, ON, Canada. Plasma protein concentrations of 1159 unique circulating biomarkers were measured using an immunoassay based on proximity extension assay (PEA) technology (Olink PEA panels, Uppsala, Sweden; 13 panels of 92 biomarkers each, including Olink Cardiovascular Disease (CVDII and CVDIII panels), Metabolism, Inflammation, OncologyII, Cardiometabolic, Organ Damage, Development, Cell Regulation, Immune response, Neurology, OncologyIII, and Neuro Exploratory). The data generated are expressed as relative quantification on the log_2_ scale of normalized protein expression (NPX) values. Although NPX values are relative quantification units, the Olink PEA platform has been extensively validated and previous work shows strong relationships between measurements from the multiplex Olink panel and singleplex assays of the same markers with absolute units (*78*). Across all 92 assays of each panel, all intra-assay and inter-assay coefficients of variation (%CV) were ≤ 30%. For each panel, the mean intra-assay %CV ranged from 5.9 to 12.9%, and the mean inter-assay %CV ranged from 8.4 to 20.2% (*79*). Individual samples were excluded on the basis of quality controls for immunoassay and detection, as well as the degree of hemolysis (*21*). NPX values were rank-based normal transformed for further analyses.

### Circulating protein level GWAS of 1159 unique biomarkers

PURE participants suitable for proteomics analyses were directly genotyped for >800,000 polymorphisms on the Thermo Fisher Scientific Axiom Precision Medicine Research Array (release 3). The genotyping quality control has been described elsewhere (*21*). Briefly, sample-level quality control checks included assessments of sample completeness (call rate > 0.95), potential sample mix-ups (discrepancies between reported versus genetically determined sex and/or ethnicity), genetic duplicates, and sample contamination (excess heterozygosity). Samples exhibiting nonambiguous discrepancies between genetic and self-reported ancestry were removed. Variant-level quality control checks included assessments of variant completeness (call rate > 0.985), plate and batch effects, non-Mendelian segregation within families (Mendelian errors), HWE deviations (HWE *P* < 1 × 10^−5^), and variant frequency (MAF > 0.001). After sample and variant quality control procedures, up to 9150 samples from Europeans, Latins, and Persians and 749,783 variants remained. The average genotyping call rate among passing samples was 0.996535. Analyses were restricted to participants from European, Latin, or Persian ancestry as LD differs between ethnic groups, and thus, might prevent MR assumption violation. We conducted a GWAS analysis (also known as pQTLs), testing the association between each of the 1159 plasma protein concentration and each of the common genetic variants in 9150 PURE participants of European, Latin, or Persian ancestry, using a linear regression model adjusted for age, sex, and 20 ancestry-specific principal components.

### Bidirectional MR and colocalization analyses

To construct the genetic instruments in forward MR, we used cis-pQTL variations that were associated with circulating protein levels (*P* < 5 × 10^−6^) in the PURE study, were located up to 200 kb downstream or upstream of the gene that codes for each measured protein, and were independent (pairwise *r*^2^ < 0.1). We chose to use cis-pQTLs derived from the GWAS of each of the 1159 circulating proteins assayed in the PURE study and did not incorporate cis-pQTLs from the UKBB to avoid bias due to sample overlap between the GWAS summary statistics used for the exposures and outcomes in subsequent two-sample MR analyses (*80*). For MR analysis, where *D*_f_ or cardiometabolic traits were used as exposures, we selected SNPs independently associated with each phenotype with *P* < 5 × 10^−6^ and *r*^2^ < 0.1. The data source for *D*_f_ was our GWAS meta-analyses, while publicly available summary statistics from consortia were used to derive the genetic instrument for the 47 cardiometabolic traits (tables S12 to S15).

As a genetic instrument linked to a protein-altering variant can influence the measurement of the protein binding affinity, genetic variations in the pleiotropic major histocompatibility complex locus, missense and splicing site variants, or variants in LD with those variants (*r*^2^ ≥ 0.9) were excluded in both forward and reverse MR analyses.

For both forward and reverse MR, the IVW method was prioritized above the MR Egger approach to estimate the associations unless the MR Egger intercept (which tests for pleiotropy) was substantially different from 0 (*P* < 0.05). Additionally, other methods, such as the weighted median, mendelian randomization using the robust adjusted profile score (MR-RAPS), and Mendelian Randomization Pleiotropy RESidual Sum and Outlier (MR-PRESSO) methods, were included. To avoid weak instrument bias, we used genetic instruments with an *F* statistic of more than 10 (*81*). A Bonferroni threshold of significance was used for the MR analysis. MR analyses have been performed using TwoSampleMR and mr.raps R packages.

MR was performed separately in three ethnic groups—European, Persian, and Latin—and the results were meta-analyzed using the fixed effect method from the metafor R package rma.uni function. Multivariable MR is a statistical method used to investigate the causal relationship between multiple exposures or risk factors and an outcome of interest (*50*). In multivariable MR, the aim is to assess the mediating role of intermediate variables in the causal pathway between an exposure and an outcome. To conduct multivariable MR analysis, genetic variants that are associated with both the exposure and/or the intermediate outcomes were used with the MVMR R package (*82*). Phenome-wide MR used similar methods to those used in forward MR, with genetic instruments being the cis-pQTLs. Genetic associations for outcomes were extracted from publicly available summary statistics of genetic consortia (tables S11 and S12).

To determine whether the causal variant underlying a significant GWAS association for *D*_f_ is shared with the biomarker pQTL, colocalization analysis was performed using the pairwise conditional analysis and colocalization analysis (PWCoCo) pipeline (*83*). We excluded the presence of colocalization when the posterior probability that an association existed between a genetic variant and both *D*_f_ and biomarker concentration (H3 + H4) was less than 80% (*83*). We complemented the colocalization with an additional analysis between pQTL biomarkers and blood sc-eQTLs from PMBCs generated by the Onek1k resource (*22*). This step was completed following the Coloc pipeline, which is very similar to that of PWCoCo. Essentially, we extracted those SNPs that colocalized in the GWAS-pQTL analysis, which were then used for an additional colocalization between sc-eQTLs and pQTLs. Causal variants colocalized in expression and proteomic levels if the posterior probability of sharing the causal signal is PPH4 > 0.8.

### Expression and chromatin activity in retinal tissue

To further support the role of associated *D*_f_ loci and cis-pQTL biomarkers in retinal tissue, we assessed the evidence of their expression levels and the activity of that genomic region using the Gene Regulatory Networks in Human Retina (Retina GRN) (*84*) and human retinal networks and enriched annotation from eyeIntegration software (*85*). Both software programs combine published gene expression data, expression quantitative trait loci (eQTL) information, and chromatin accessibility and loops data, in the case of Retina GRN, from human retinal tissue to derive the regulatory networks.

### Protein-protein interaction network analysis of MR biomarkers

We evaluated the biological mechanisms and the interactions between associated MR biomarkers through a protein-protein interaction network study and an enrichment analysis. We used biomarkers that had *P* < 0.001 for each MR analysis in European, European-Latin, or European-Latin-Persian populations to investigate the general processes. STRING (*86*) and DAVID (*87*) software were then used to identify the protein-protein interactions of MR biomarkers and their genetic and pathway enrichment. Enriched pathways and functional annotations were considered if FDR and Bonferroni correction were < 0.01.
