# Supplementary material for: Mendelian randomization study implicates inflammaging biomarkers in retinal vasculature, cardiovascular diseases, and longevity
Source: Sci Adv. 2025 Oct 24;11(43):eadu1985. doi: 10.1126/sciadv.adu1985 (PMC12551708; doi:10.1126/sciadv.adu1985)
Supplement: Supplementary file 1 — Figs. S1 to S3 Legends for tables S1 to S20 [file sciadv.adu1985_sm.pdf]

Science Advances
AAAS

Supplementary Materials for

Mendelian randomization study implicates inflammaging biomarkers in retinal vasculature, cardiovascular diseases, and longevity

Ana Villaplana-Velasco *et al.*

Corresponding author: Erola Pairo-Castineira, erola.pairo@regeneron.com; Marie Pigeyre, pigeyrem@mcmaster.ca

Sci. Adv. **11**, eadu1985 (2025)
DOI: 10.1126/sciadv.adu1985

The PDF file includes:

Figs. S1 to S3
Legends for tables S1 to S20

Other Supplementary Material for this manuscript includes the following:

Tables S1 to S20

Figure S1: QQ plots of the GWAS meta-analysis for D_f (CLSA-GODARTS-UKB)

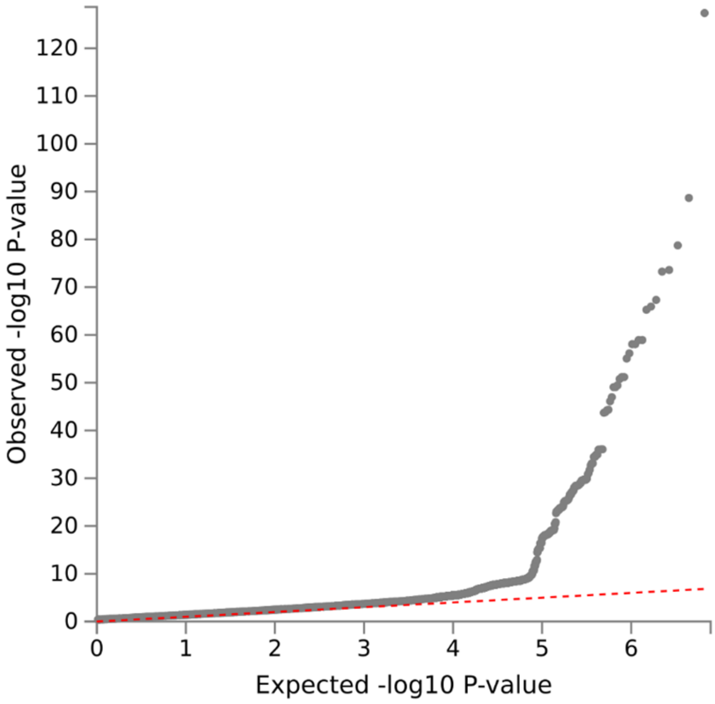

Figure S2. Forest plot of the genetic variant *HERC2 rs12913832* effect on D_f in the GWAS meta-analysis models

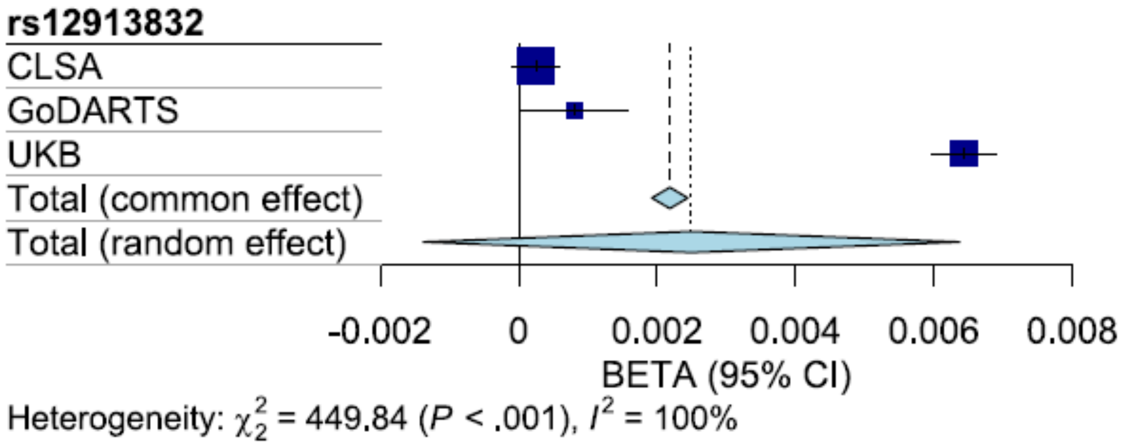

Figure S3: Colocalization of FCGR2B pQTL and sc-eQTL in peripheral blood mononuclear cells (PMBCs)

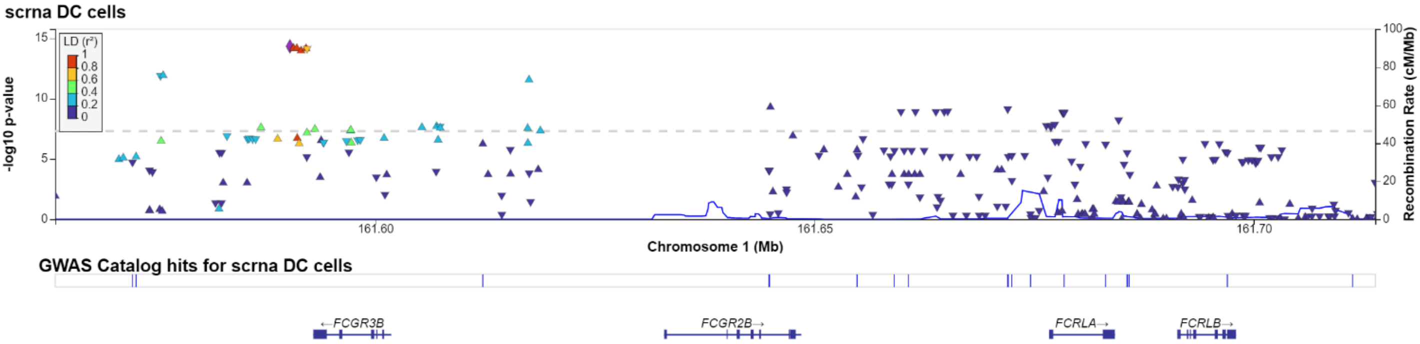

Abbreviations: scRNA DC: Single-cell RNA sequencing of dendritic cells

Supplemental Tables

Table S1: Demographic characteristics of CLSA participants
Table S2: Demographic characteristics of UKB participants
Table S3: Demographic characteristics of GODARTS participants
Table S4: Suggestive associations (P<10-6) of the GWAS meta-analysis for retinal Df from the CLSA-GODARTS-UKBB cohorts
Table S5: MAGMA gene analyses of GWAS meta-analysis of Df
Table S6: MAGMA pathway analyses of GWAS meta-analysis of Df
Table S7: Genetic correlations between Df and cardiovascular traits and longevity
Table S8: Two-Sample forward MR results of biomarkers on Df
Table S9: Two-Sample forward MR results of biomarkers on Df, stratifed by diabetes status (individuals without diabetes)
Table S10: Two-Sample forward MR results of biomarkers on Df, stratified by diabetes status (individuals with diabetes)
Table S11: Two-Sample reverse MR results of Df on biomarkers
Table S12: Two-Sample forward MR results of Df-related biomarkers on cardiometabolic outcomes
Table S13: Significant Two-Sample forward MR results of Df-related biomarkers on cardiometabolic outcomes
Table S14: Two-Sample forward MR results of cardiometabolic traits on Df
Table S15: Two-Sample reverse MR results of Df on cardiometabolic outcomes
Table S16: Colocalization of Df SNPs with biomarkers pQTLs
Table S17: Colocalization of FCGR2B pQTLs and sc-eQTL in peripheral blood mononuclear cells (PMBCs)
Table S18: STRING enrichment analyses of MR biomarkers associated with Df (P-value <0.001)
Table S19: DAVID enrichment analyses of MR biomarkers associated with Df (P-value <0.001)
Table S20: Expression and chromatin activity analysis on retinal tissue for the biomarkers identified for Df (P-value <0.0001) in the MR analyses
